# Occasions, Locations, and Reasons for Consuming Sugar-Sweetened Beverages among U.S. Adults

**DOI:** 10.3390/nu15040920

**Published:** 2023-02-12

**Authors:** Seung Hee Lee, Sohyun Park, Thomas C. Lehman, Rebecca Ledsky, Heidi M. Blanck

**Affiliations:** 1 Division of Nutrition, Physical Activity, and Obesity, National Center for Chronic Disease Prevention and Health Promotion, Centers for Disease Control and Prevention, Atlanta, GA 30341, USA; 2 FHI 360, Social Marketing and Communication, Washington, DC 20009, USA

**Keywords:** sugar-sweetened beverage, adult, dietary intake, food choice

## Abstract

Frequent intake of sugar-sweetened beverages (SSBs) is associated with adverse health outcomes such as obesity, type 2 diabetes, and cardiovascular disease. Little is known about when, where, and why U.S. adults consume SSBs. This study, using data from an online survey distributed in 2021, examined the occasions, locations, and reasons for consuming SSBs and the characteristics of the adults who consume them. Nearly 7 of 10 adults reported consuming a SSB (1–6 times) in the past 7 days, and more than a third (38%) reported doing so once or more per day (on average). For comparative purposes, the sample was limited to adults who reported consuming SSBs within the last 7 days. Mealtimes were reported as the most frequent occasion for the intake of SSBs (43%) and SSBs were most often consumed at home (70%). Over half of respondents (56%) reported they consume SSBs because they enjoy the taste. Younger adults (18–34 years old) were more likely to consume SSBs in social settings than older adults (≥50 years old). Hispanic adults were less likely to consume SSBs at the beginning of the day compared to non-Hispanic White adults. Younger (18–34 years old) and middle-aged (35–49 years old) adults were more likely to consume SSBs in restaurants, at work, and in cars than older adults (≥50 years old). Women were less likely to consume SSBs at work than men. Hispanic adults were less likely to consume SSBs in cars than non-Hispanic White adults, while those earning USD 50,000–<USD 100,000 were more likely to consume SSBs in cars than those earning ≥USD 100,000. Younger and middle-aged adults were more likely to consume SSBs due to cravings and enjoyment of the carbonation compared to older adults. These findings provide insights on specific populations for whom to tailor messaging and adapt interventions to help reduce SSB intake.

## 1. Introduction 

Sugar-sweetened beverages (SSBs) are the largest sources of added sugars in the U.S. adult diet and include carbonated and non-carbonated soft drinks, fruit drinks, sports drinks, energy drinks, sweetened water, and sweetened coffee/tea drinks that contain added sugars [1]. Excess intake of SSBs is associated with adverse health outcomes such as weight gain and the risk of type 2 diabetes, cardiovascular diseases, and related risk factors [2,3,4,5]. While the consumption of SSBs has decreased over the past decades, SSB intake among U.S. adults remains high [6]. A study reported that 63% of U.S. adults drank SSBs at least once per day in 2010 and 2015 [7]. National Health and Nutrition Examination Survey (NHANES) data between 2011 and 2014 showed that U.S. adults consumed an average of 145 kcal/day of SSBs, corresponding to 6.5% of the total calories with higher intake levels reported among younger age groups and among non-Hispanic Black and Hispanic men and women [8]. There is an abundant body of evidence regarding the high prevalence of SSB intake and sociodemographic characteristics associated with SSB intake among U.S. adults [6,9,10]. However, little is known about when, where, and why individuals consume SSBs with reference to a national data set. While several studies examined eating occasions, locations, and reasons for consuming SSBs, they only included limited response options, were based on older data, and/or had small sample sizes [11,12,13,14,15]. Understanding defined eating occasions, locations, and reasons that influence SSB intake could aid the design of future communication campaigns to decrease SSB intake among U.S. adults. Therefore, the purposes of our study were to describe the eating occasions, locations, and reasons for consuming SSBs among U.S. adults and to explore associations between the outcome variables (i.e., eating occasions, locations, and reasons for consuming SSBs) and sociodemographic and other characteristics among SSB consumers. 

## 2. Methods 

### 2.1. Sample and Survey Administration 

A cross-sectional study was conducted using the Ipsos G&A Omnibus Survey 2021, a nationally representative sample of U.S. adults, via KnowledgePanel^®^, an online research panel [16]. KnowledgePanel’s recruitment process employs an address-based sampling methodology employing the latest Delivery Sequence Files of the USPS, which is a database with full coverage of all delivery points in the US. Households invited to join the panel were randomly selected from all available households in the US, and persons in the sampled households were invited to join and participate in the panel. A tablet and internet connection were provided (if needed). Participants received unique password-protected log-in information used to complete online surveys. In 2021, the Omnibus survey was sent to 1750 adults on the panel and 1013 adults completed the survey, yielding a response rate of 58%. For subset analyses that explored the occasions, locations, and reasons for consuming SSBs, we limited samples to adults who reported consuming any SSBs during the past 7 days (*n* = 658). All sampled adults received an invitational message from Ipsos with a link to an IRB-approved study information sheet. Those who consented to participate could then proceed to the online survey.

### 2.2. Measures

Outcome variables defined in this study were occasions, locations, and reasons for consuming SSBs on a typical day. Regarding the occasions of SSB intake, we asked the following question: “When do you drink sugary drinks? Check all that apply.” There were 10 responses: (1) at the beginning of the day, (2) at mealtime, (3) between meals/when snacking, (4) at the end of the day, (5) during or after exercising or being physically active, (6) when commuting, (7) for a special event or celebration, (8) for a special meal with family or friends, (9) in social settings/when others are drinking these drinks, and (10) none of the above. 

Regarding the locations of SSB intake, we asked the following question: “Where do you drink sugary drinks? Check all that apply.” There were 6 responses: (1) at home; (2) at restaurants/bars; (3) in the car; (4) at work; (5) in parks, gyms, or other recreation areas; and (6) none of the above. 

With regard to the reasons for SSB intake, we asked the following question: “Which of the following are reasons why you drink sugary drinks? Check all that apply.” There were 14 responses preceded by the prompt, “I drink sugary drinks because…”: (1) I enjoy the taste, (2) they make me feel happy, (3) they give me energy, (4) they make my meal better, (5) they are an alternative to a food snack, (6) they satisfy my thirst, (7) they are a habit/part of my routine, (8) they are easy to carry/transport, (9) they are convenient to drink, (10) they are easy to find/buy, (11) I like the carbonation/fizz/bubbles, (12) they are inexpensive/affordable, (13) they satisfy my cravings for something sweet, and (14) none of the above. 

SSB intake was determined by the following five questions: (1) “During the past 7 days, how many times did you drink REGULAR SODA or POP? These are drinks that contain added sugars, such as Coke, Pepsi, or Sprite.”; (2) “During the past 7 days, how many times did you drink SPORTS or ENERGY DRINKS? These are drinks that contain added sugars such as Gatorade, Red Bull, Monster, and Vitamin Water.”; (3) “During the past 7 days, how many times did you drink SWEETENED FRUIT DRINKS? These are drinks that contain added sugars such as Kool-Aid, fruit punch, cranberry, and lemonade. Include fruit drinks you made at home and added sugars to such as homemade lemonade.”; (4) “During the past 7 days, how many times did you drink SWEETENED COFFEE or TEA? These include drinks that are served hot or cold and drinks that are purchased in cups, cans, or bottles. They include presweetened tea and coffee drinks such as Arizona Iced Tea and Starbucks Frappuccino. These drinks also include coffee and tea drinks you sweetened yourself by adding sugar or honey.”; and (5) “During the past 7 days, how many times did you have a DRINK WITH A SWEETENED MIXER? These include drinks sweetened with mixers such as regular soda or pop, energy drinks, tonic water, simple syrup, or sweetened fruit drinks like cranberry juice cocktail.” The response options for each question were none, 1–3 times/week, 4–6 times/week, 1 time/day, 2 times/day, 3 times/day, and ≥4 times/day. To estimate weekly intake, 1–3 times/week was converted to 2 times/week, 4–6 times/week was converted to 5 times/week, and ≥4 times/day was converted to 4 times/week. To calculate the total frequency of daily SSB intake, we added the responses from five SSB questions.

Sociodemographic variables assessed included age (18–34 years old, 35–49 years old, and ≥50 years old), gender, race/ethnicity (non-Hispanic (NH) Black, Hispanic, NH other, and NH White), education level (≤high school, some college, and college graduate), annual household income (<USD 25,000, USD 35,000–USD 74,999, USD 75,000–USD 99,999, or ≥USD 100,000), and marital status (married/domestic partnership and not married). Not married comprised widowed, divorced, separated, or never married. Other covariates included weight status, census region (Northeast, Midwest, South, or West) [17], having children aged < 18 years in the household (yes or no), and self-identified urbanicity (urban, rural, or suburb). BMI was calculated using self-reported weight and height data, and weight status was grouped into underweight/healthy weight (BMI < 25 kg/m^2^), overweight (BMI 25–<30 kg/m^2^), or obesity (BMI ≥ 30 kg/m^2^) [18].

### 2.3. Statistical Analyses

For unadjusted bivariate analyses, descriptive statistics were used to examine sociodemographic characteristics associated with SSB intake as well as occasions, locations, and reasons for consuming SSBs using chi-square tests. In this study, *p* value < 0.05 is considered as statistically significant. For adjusted analyses, we used a total of 12 multivariate logistic regression models to calculate adjusted odds ratios and 95% confidence intervals (Cis) for sociodemographic characteristics associated with four most frequent responses according to occasions, locations, and reasons. Each model included all sociodemographic characteristics. We used survey procedures to account for sampling weights and sampling design using SAS 9.4 (SAS Institute Inc., Cary, NC, USA). 

## 3. Results

Nearly 7 of 10 of the surveyed adults reported consuming any SSB (1–6 times) in the past 7 days, and 38% reported doing so at least 7 times during the past 7 days (on average 1 or more times per day). The prevalence of SSB intake more than seven times per week was the highest among adults aged 35–49 years old, NH Black and Hispanic adults, those with ≤high school education, those with the lowest household income (<USD 25,000/year), and with children (<18 years old) in the household (*p* < 0.05 based on χ^2^ tests) (Table 1).

**Figure 1:**
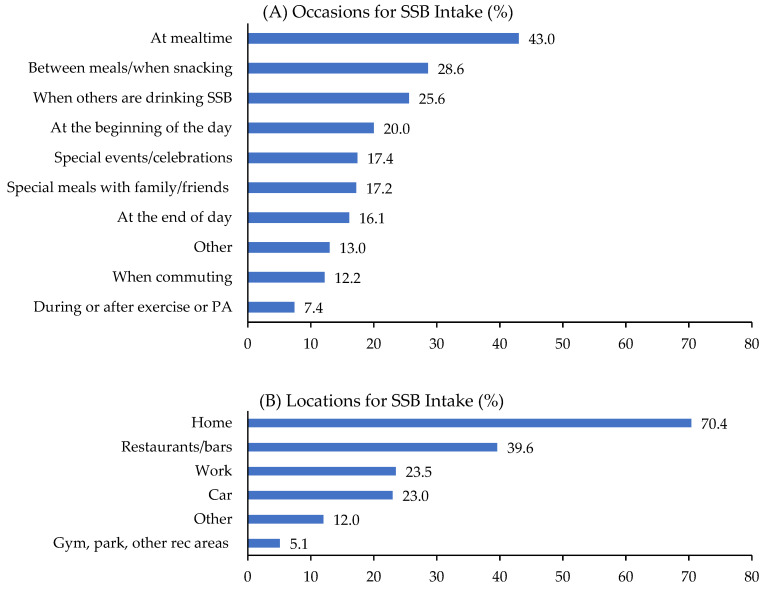

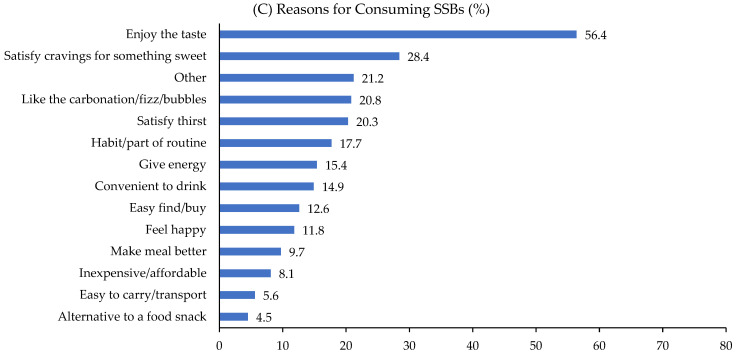
(**A**) Occasions, (**B**) locations, and (**C**) reasons for consuming sugar-sweetened beverages (SSBs) among U.S. adults who reported consuming SSBs during the past 7 days (*n* = 658) (Omnibus Survey, 2021).

Overall, occasions for which SSBs were consumed on a typical day were highest at mealtime (43%), followed by between meals/when snacking (29%), in social settings/when others are drinking SSBs (26%), at the beginning of the day (20%), at special events/celebrations (17%), at special meals with family or friends (17%), at the end of the day (16%), and when commuting (12%). The locations where SSBs were consumed most were at home (70%), followed by restaurants/bars (40%), work (24%), and in the car (23%). The most common reasons for drinking SSBs were enjoying the taste (56%), satisfying cravings for something sweet (28%), liking the carbonation (21%), and satisfying thirst (20%) (Figure 1).

Of those who reported consuming SSBs during the past 7 days (*n* = 658), the prevalence of the top four occasions, locations, and reasons for SSB intake significantly varied by certain characteristics. In unadjusted bivariate analysis of the top four occasions for drinking SSBs (i.e., at mealtime, between meals/snacking, in social settings, and at the beginning of the day), significant differences were observed according to age, marital status, and weight status regarding the consumption of SSBs in a social setting and according to race/ethnicity concerning consumption at the beginning of the day (Table 2). Based on multivariable logistic regression analysis, younger adults had higher odds of consuming SSBs in social settings (18–34 years old, AOR:2.1, and 95% CI: 1.2–3.6) than older adults (≥50 years old); Hispanic adults had lower odds of consuming SSBs between meals/snacking (AOR:0.4; 95% CI: 0.2–0.9) or at the beginning of the day (AOR:0.3; 95% CI: 0.1–0.6) than NH White adults (Table 2).

In an unadjusted bivariate analysis of the top four locations in which SSBs were consumed (i.e., home, restaurant, work, and car), significant differences were present according to age concerning the consumption of SSBs in restaurants; according to age, gender, and having children in the household with respect to the consumption of SSBs at work; and according to age, race/ethnicity, annual household income, and having children in the household with regard to the consumption of SSBs in a car (Table 3). Based on multivariate logistic regression analysis, younger (18–34 years old) and middle-aged adults (35–49 years old) had higher odds of consuming SSBs in restaurants (AOR:1.9, 95% CI: 1.2–3.2; AOR:2.3, and 95% CI: 1.4–3.7, respectively), at work (AOR: 2.9 and 95% CI: 1.6–5.2; AOR:2.7 and 95% CI: 1.5–4.9), and in cars (AOR:2.0 and 95% CI: 1.1–3.7; AOR:3.1 and 95% CI: 1.7–5.6) than older adults (≥50 years old). Women were less likely to consume SSBs at work (AOR:0.6 and 95% CI: 0.4–0.9) than men. Hispanic adults were less likely to consume SSBs in cars (AOR:0.3 and 95% CI: 0.1–0.6) than NH White adults, while those earning USD 50,000–<USD 100,000 were more likely to consume SSBs in cars (AOR:2.3 and 95% CI: 1.4–4.1) than those earning ≥USD 100,000 (Table 3).

In an unadjusted bivariate analysis, of the top four reasons (i.e., taste, craving, fizz, and thirst) for consuming SSBs, significant differences existed according to annual household income with respect to the consumption of SSBs because of taste, and according to age with respect to the consumption of SSBs because of craving or fizz (Table 4). Based on multivariate logistic regression analysis, younger adults and middle-aged adults had almost three times higher odds of consuming SSBs due to cravings (AOR:2.8, 95% CI: 1.7–4.8; AOR:2.8, 95% CI: 1.7–4.9) or because they like the fizz/bubble (AOR:2.4, 95% CI: 1.4–4.1; AOR:2.1, 95% CI: 1.2–3.7) than older adults (Table 4). 

## 4. Discussion 

Overall, nearly 7 of 10 of the surveyed U.S. adults consumed any SSB in the past week, and 38% reported doing so at least 7 times during the past week (or on average once a day) in 2021. The daily intake of SSBs in our study was lower than previous findings in which it was shown that 63% of U.S. adults in 2010 and 2015 consumed SSBs at least once a day [7], with significant differences in sociodemographic characteristics. While most adults reported drinking SSBs at least one time in the previous seven days, the groups with the highest levels of consumption include adults aged 35–49 years old, NH Black and Hispanic adults, those with ≤high school education, those with low household income (<USD 25,000/year), and those who have children (<18 years old) in their household.

In our study, the four most common occasions at which SSBs were consumed among U.S. adults were (1) at mealtime, (2) between meals/when snacking, (3) in social settings/when others were drinking SSBs, and (4) at the beginning of the day. Similar to our findings, a previous study showed that adults consumed 85 kcal and 66 kcal from SSBs during mealtime and for snacks, respectively [11]. We also found that the top four leading locations in which SSBs were consumed were (1) at home, (2) in restaurants/bars, (3) at work, and (4) in cars. In accordance with our findings, previous studies reported that more than half of the calories obtained from SSBs were consumed at home among U.S. adults [12,19]. Based on 2011–2012 NHANES data, SSB consumers purchased 52% of their SSB-derived calories from supermarkets/grocery stores, 16% from fast-food restaurants, 11% from convenience stores, 8% from full-service restaurants, and 4% from vending machines [20]. It is possible that U.S. adults purchase most of their SSBs from supermarkets/grocery stores and consume SSBs at home. In our study, the top four leading reasons for drinking SSBs among U.S. adults were (1) enjoying the taste, (2) satisfying cravings for something sweet, (3) liking the carbonation, and (4) satisfying thirst. Another study also reported that the taste is one of the most important driving factors of the consumption of SSBs [13].

While communication campaigns have been demonstrated to reduce SSB sales and consumption among adults [21,22,23]. the findings from this research provide additional insights into opportunities for identifying an audience, messaging, and SSB counter-marketing. SSB counter-marketing messages can be placed in locations where SSBs are likely to be purchased (i.e., grocery stores) and consumed (i.e., home). Geofencing or location-based marketing, wherein virtual boundaries are established around a location and a digital notification or advertisement is prompted when a mobile device crosses these boundaries, can be used in grocery stores and select neighborhoods with high SSB consumption [24]. In addition, since SSB consumption is highest at mealtime, SSB counter-marketing messages can be sent through channels likely to be viewed in the home (e.g., internet, streaming media, radio, and television) [25]. Such placements can be concentrated at times when adults are most likely to be making decisions regarding beverage choices associated with meals, and through channels they are most likely to be using at these times (e.g., mobile and tablet recipe applications, YouTube food channels, etc.). 

Among the adults who drank any SSBs during the past 7 days, most said they did so because they enjoy the taste. Future research could explore the taste appeal of healthy alternatives to SSBs and the effectiveness of messaging (e.g., counter-marketing) that prioritizes the benefits of not drinking SSBs over the appeal of taste. 

## 5. Conclusions

This study is one of the first studies examining when, where, and why American adults consume SSBs using nationally representative data. However, this study is subject to at least two limitations. First, it is a cross-sectional study and, therefore, causal relationships cannot be determined. Second, the outcome of SSB intake was measured by frequency; thus, the volume of intake was not measured. Third, the questionnaire used in the study was not validated. Lastly, it is possible that the COVID-19 pandemic might have had an impact on the responses to the occasions, locations, and reasons for consuming SSBs. In conclusion, however, the findings from this study can help public health programs, educators, dieticians, and others that engage with individuals and families to consider producing messaging information that may help focused populations reduce SSB intake to support their health. 

## Figures and Tables

**Table 1:** Characteristics of survey respondents and their associations with sugar-sweetened beverage intake during the past 7 days among U.S. adults (Omnibus Survey 2021).

Characteristics	All Participants	Sugar-Sweetened Beverage Intake (% ± Standard Error)			
	%	0 Times/Day	1–6 Times in the Past Week	7 or More Times in the Past Week	*p* Value *
**Total (*N* = 1013)**	100	31.4 ± 1.5	31.2 ± 1.6	37.5 ± 1.6	
**Age**					
18–34 years	27.0	23.2 ± 3.1	36.6 ± 3.6	40.1 ± 3.7	0.0002
35–49 years	22.9	25.1 ± 3.0	31.2 ± 3.3	43.7 ± 3.5	
≥50 years	50.1	38.7 ± 2.0	28.2 ± 1.9	33.2 ± 2.0	
**Gender**					
Men	48.4	30.6 ± 2.1	29.9 ± 2.2	39.5 ± 2.3	0.44
Women	51.6	32.2 ± 2.1	32.3 ± 2.2	35.5 ± 2.3	
**Race/Ethnicity**					
Black, non-Hispanic	11.7	31.4 ± 5.1	23.3 ± 4.5	45.3 ± 5.4	<0.0001
Hispanic, non-Hispanic	16.5	14.1 ± 3.3	40.4 ± 4.8	45.6 ± 4.8	
Other/multiracial, non-Hispanic	8.7	28.7 ± 5.2	39.1 ± 5.6	32.1 ± 5.5	
White, non-Hispanic	63.1	36.3 ± 1.8	29.1 ± 1.8	34.6 ± 1.9	
**Education**					
≤High school	38.6	27.6 ± 2.5	27.4 ± 2.6	45.1 ± 2.8	0.001
Some college	30.1	29.4 ± 2.7	34.1 ± 2.9	36.5 ± 3.0	
College graduate	31.4	38.0 ± 2.6	33.0 ± 2.6	29.0 ± 2.5	
**Annual household income**					
<USD 25,000	12.5	28.8 ± 4.6	21.7 ± 4.2	49.5 ± 5.4	<0.0001
USD 25,000–<USD 50,000	17.5	24.7 ± 3.2	28.1 ± 3.5	47.1 ± 3.8	
USD 50,000–<USD 100,000	31.5	27.1 ± 2.6	31.6 ± 2.9	41.3 ± 3.0	
≥USD 100,000	38.5	38.8 ± 2.5	35.3 ± 2.5	26.0 ± 2.2	
**Marital status**					
Not married	41.0	29.2 ± 2.5	29.9 ± 2.6	41.0 ± 2.8	0.20
Married	59.0	33.0 ± 1.9	32.0 ± 1.9	35.0 ± 2.0	
**Weight status ^a^ (*n* = 933)**					
Underweight/healthy weight	33.4	32.5 ± 2.8	30.0 ± 2.9	37.5 ± 3.0	0.38
Overweight	31.8	36.1 ± 2.8	30.5 ± 2.8	33.4 ± 2.8	
Obesity	34.8	28.7 ± 2.6	34.3 ± 2.8	37.0 ± 2.9	
**Census region**					
Northeast	17.2	26.7 ± 3.4	32.7 ± 3.7	40.7 ± 4.0	0.31
Midwest	20.7	36.0 ± 3.4	24.5 ± 3.1	39.4 ± 3.5	
South	38.2	31.6 ± 2.5	32.0 ± 2.6	36.3 ± 2.7	
West	24.0	30.4 ± 3.1	34.3 ± 3.4	35.2 ± 3.3	
**Children present (<18 years old) in household**					
No	69.9	34.9 ± 1.8	30.8 ± 1.8	34.4 ± 1.9	0.002
Yes	30.1	23.3 ± 2.6	32.0 ± 3.0	44.7 ± 3.2	
**Urbanicity (*n* = 1011)**					0.66
Urban	31.5	31.8 ± 2.7	29.5 ± 2.7	38.7 ± 2.9	
Rural	19.0	35.4 ± 3.5	30.8 ± 3.4	33.8 ± 3.5	
Suburb	49.5	29.8 ± 2.1	31.9 ± 2.3	38.3 ± 2.4	

* Based on chi-squares tests across categories. ^a^ Weight status was based on calculated body mass index (BMI) (kg/m^2^): underweight/healthy weight, BMI < 25; overweight, BMI 25–<30; obesity, BMI ≥ 30.

**Table 2:** Unadjusted (bivariate analysis) and adjusted (multivariate analysis) relationship between top four eating occasions when sugar-sweetened beverages (SSBs) were consumed according to sociodemographic variables among U.S. adult SSB consumers—Omnibus Survey, 2021.

Characteristics	Eating Occasions When SSBs Were Consumed							
	Bivariate Analysis Prevalence (%)				Multivariate Logistic Regression Analysis Adjusted Odds Ratios (95% CI)			
	At Mealtime	Between Meals/Snacking	Social Settings	Beginning of the Day	At Mealtime	Between Meals/Snacking	Social Settings	Beginning of the Day
**Age**								
18–34 years old	44.5	31.7	33.8 *	20.3	1. 2 (0.7, 1.9)	1.2 (0.7, 2.1)	2.1 (1.2, 3.6)	1.1 (0.6, 1.9)
35–49 years old	47.3	29.8	27.8 *	25.3	1.3 (0.8, 2.1)	1.4 (0.8, 2.3)	1.8 (1.0, 3.2)	1.5 (0.9, 2.8)
≥50 years old	39.5	25.8	18.7 *	16.7	Referent	Referent	Referent	Referent
**Gender**								
Men	44.1	30.6	22.4	18.9	Referent	Referent	Referent	Referent
Women	41.8	26.6	28.7	21.0	0.9 (0.6, 1.3)	0.8 (0.5, 1.2)	1.2 (0.8, 1.9)	1.3 (0.8, 2.0)
**Race/Ethnicity**								
Black, non-Hispanic	40.0	39.4	27.0	12.0 *	1.0 (0.6, 1.9)	1.7 (0.9, 3.1)	0.8 (0.4, 1.6)	0.5 (0.2, 1.1)
Hispanic	50.1	20.2	24.4	9.5 *	1.3 (0.8, 2.3)	0.4 (0.2, 0.9)	0.9 (0.5, 1.8)	0.3 (0.1, 0.6)
Other/multiracial, non-Hispanic	39.0	25.6	27.2	20.4 *	0.8 (0.4, 1.6)	0.9 (0.4, 2.0)	0.9 (0.4, 1.8)	0.7 (0.3, 1.4)
White non-Hispanic	41.7	29.8	25.5	25.2 *	Referent	Referent	Referent	Referent
**Education**								
≤High school	44.8	33.4	25.2	19.9	1.2 (0.7, 1.9)	1.6 (0.9, 2.7)	0.7 (0.4, 1.3)	1.3 (0.7, 2.2)
Some college	47.0	27.5	25.0	19.8	1.3 (0.8, 2.1)	1.1 (0.6, 1.8)	1.0 (0.6, 1.6)	1.2 (0.7, 2.1)
College graduate	35.8	22.9	26.8	20.2	Referent	Referent	Referent	Referent
**Annual household income**								
<USD 25,000	41.7	31.6	26.4	23.3	1.2 (0.6, 2.4)	1.1 (0.5, 2.5)	1.3 (0.6, 2.7)	1.3 (0.5, 3.0)
USD 25,000–<USD 50,000	46.1	31.2	21.9	18.2	1.2 (0.7, 2.1)	1.3 (0.7, 2.4)	0.8 (0.4, 1.6)	0.7 (0.4, 1.4)
USD 50,000–<USD 100,000	43.9	29.7	29.0	19.6	1.3 (0.8, 2.0)	1.4 (0.8, 2.2)	1.4 (0.8, 2.3)	0.9 (0.5, 1.5)
≥USD 100,000	40.8	24.9	23.9	20.1	Referent	Referent	Referent	Referent
**Marital status**								
Not married	44.2	32.2	30.4 *	21.0	1.1 (0.8, 1.7)	1.2 (0.8, 1.9)	1.4 (0.8, 2.3)	1.1 (0.7, 1.8)
Married	42.0	26.0	22.1 *	19.2	Referent	Referent	Referent	Referent
**Weight status ^a^ (*n* = 602)**								
Underweight/healthy weight	39.1	26	26.8 *	22.7	Referent	Referent	Referent	Referent
Overweight	47.6	28.3	15.9 *	18.7	1.4 (0.9, 2.2)	1.1 (0.7, 1.9)	0.6 (0.3, 1.1)	0.8 (0.5, 1.4)
Obesity	42.9	30.6	31.3 *	19.3	1.1 (0.7, 1.8)	1.3 (0.8, 2.0)	1.5 (0.9, 2.5)	0.8 (0.5, 1.4)
**Census region**								
Northeast	42.4	23.7	31.2	16	1.3 (0.8, 2.2)	0.7 (0.4, 1.2)	1.4 (0.8, 2.6)	0.7 (0.4, 1.5)
Midwest	42.6	28.5	27.8	27.2	1.2 (0.7, 1.9)	0.8 (0.5, 1.3)	1.2 (0.6, 2.1)	1.3 (0.8, 2.4)
South	41.2	33.0	22.3	19.1	Referent	Referent	Referent	Referent
West	46.4	25.6	24.7	18.7	1.1 (0.7, 1.9)	0.8 (0.5, 1.5)	0.9 (0.5, 1.6)	1.2 (0.7, 2.2)
**Having children (<18 years old) in household**								
No	40.7	28.2	24.1	19.2	Referent	Referent	Referent	Referent
Yes	47.3	29.4	28.6	21.4	1.1 (0.7, 1.7)	0.9 (0.6, 1.5)	1.0 (0.6, 1.7)	1.1 (0.6, 1.8)
**Urbanicity**								
Urban	41.7	27.6	28.4	19.5	Referent	Referent	Referent	Referent
Rural	40.2	28.2	19.6	20.2	1.0 (0.6, 1.7)	1.3 (0.7, 2.3)	0.5 (0.3, 1.0)	1.7 (0.4, 1.4)
Suburban	44.5	29.6	25.6	20.4	1.1 (0.8, 1.7)	1.6 (1.0, 2.6)	0.7 (0.5, 1.1)	0.9 (0.5, 1.5)

* *p* < 0.05 across categories based on chi-squares tests. ^a^ Weight status was based on calculated body mass index (BMI) (kg/m^2^): underweight/healthy weight, BMI < 25; overweight, BMI 25–<30; obesity, BMI ≥ 30.

**Table 3:** Unadjusted (bivariate analysis) and adjusted (multivariate analysis) relationship between top four locations where sugar-sweetened beverages (SSBs) are consumed according to sociodemographic variables among U.S. adult SSB consumers—Omnibus Survey, 2021.

Characteristics	Locations Where SSBs Are Consumed							
	Bivariate Analysis Prevalence (%)				Multivariate Logistic Regression Analysis Adjusted Odds Ratios (95% CI)			
	Home	Restaurant	Work	Car	Home	Restaurant	Work	Car
**Age**								
18–34 years old	68.2	44.9 *	30.2 *	26.8 *	0.9 (0.6, 1.5)	1.9 (1.2, 3.2)	2.9 (1.6, 5.2)	2.0 (1.1, 3.7)
35–49 years old	74.1	47.9 *	31.7 *	33.5 *	1.5 (0.9, 2.5)	2.3 (1.4, 3.7)	2.7 (1.5, 4.9)	3.1 (1.7, 5.6)
≥50 years old	70.2	31.3 *	14.2 *	14.5 *	Referent	Referent	Referent	Referent
**Gender**								
Men	72.9	39.1	27.9 *	24.3	Referent	Referent	Referent	Referent
Women	68.3	40.0	19.2 *	21.8	0.8 (0.5, 1.2)	1.0 (0.7, 1.4)	0.6 (0.4, 0.9)	0.9 (0.6, 1.4)
**Race/Ethnicity**								
Black, non-Hispanic	75.1	31.5	21.5	24.8 *	1.2 (0.6, 2.4)	0.5 (0.3, 1.0)	1.0 (0.5, 2.0)	0.8 (0.4, 1.9)
Hispanic	65.7	42.7	23.4	12.9 *	0.7 (0.4, 1.3)	1.2 (0.7, 2.2)	0.9 (0.4, 1.7)	0.3 (0.1, 0.6)
Other/multiracial, non-Hispanic	66.1	38.9	23.4	19.2 *	0.8 (0.4, 1.6)	0.9 (0.5, 1.8)	1.1 (0.5, 2.3)	0.5 (0.2, 1.3)
White, non-Hispanic	72.0	40.2	23.9	26.8 *	Referent	Referent	Referent	Referent
**Education**								
≤High school	74.6	39.0	24.6	24.7	1.1 (0.6, 1.8)	0.8 (0.5, 1.3)	1.3 (0.7, 2.3)	1.2 (0.6, 2.2)
Some college	66.9	41.7	24.3	24.5	0.7 (0.4, 1.2)	1.2 (0.7, 1.9)	1.3 (0.7, 2.3)	1.4 (0.7, 2.5)
College graduate	68.7	38.2	21.0	19.1	Referent	Referent	Referent	Referent
**Annual household income**								
<USD 25,000	71.7	35.4	17.8	27.8 *	1.2 (0.5, 2.7)	0.9 (0.4, 1.8)	0.5 (0.2, 1.3)	1.6 (0.7, 3.9)
USD 25,000–<USD 50,000	75.3	36.8	25.5	16.4 *	1.4 (0.8, 2.6)	1.0 (0.6, 1.8)	1.0 (0.5, 2.0)	0.9 (0.4, 1.8)
USD 50,000–<USD 100,000	67.9	44.0	24.3	30.3 *	1.1 (0.9, 2.2)	1.3 (0.8, 2.1)	1.0 (0.6, 1.6)	2.3 (1.4, 4.1)
≥USD 100,000	70.1	38.4	23.7	17.8 *	Referent	Referent	Referent	Referent
**Marital status**								
Not married	72.8	40.9	24.3	25.5	1.4 (0.9, 2.2)	1.1 (0.7, 1.7)	1.1 (0.6, 1.8)	1.4 (0.8, 2.4)
Married	68.9	38.7	22.9	21.3	Referent	Referent	Referent	Referent
**Weight status ^a^ (*n* = 602)**								
Underweight/healthy weight	67.4	36.1	19.4	21.4	Referent	Referent	Referent	Referent
Overweight	72.4	36.3	24.8	18.4	1.2 (0.7, 1.9)	1.1 (0.7, 1.8)	1.5 (0.8, 2.6)	0.9 (0.5, 1.7)
Obesity	70.3	44.5	25.3	26.9	1.1 (0.7, 1.8)	1.6 (1.0, 2.6)	1.6 (0.9, 2.8)	1.6 (0.9, 2.8)
**Census region**								
Northeast	70.4	43.7	23.3	25.3	0.9 (0.5, 1.5)	1.3 (0.7, 2.2)	1.1 (0.6, 2.0)	1.2 (0.6, 2.2)
Midwest	73.3	45.6	28.0	25.1	1.1 (0.7, 1.9)	1.2 (0.7, 2.0)	1.2 (0.6, 2.1)	0.8 (0.4, 1.5)
South	71.7	37.0	24.2	21.9	Referent	Referent	Referent	Referent
West	66.7	35.7	18.9	21.4	0.9 (0.5, 1.6)	0.7 (0.4, 1.1)	0.6 (0.3, 1.1)	1.2 (0.6, 2.2)
**Having children (<18 years old) in household**								
No	72.2	36.9	19.8 *	19.0 *	Referent	Referent	Referent	Referent
Yes	67.3	45.0	30.8 *	31.0 *	0.6 (0.4, 1.0)	1.0 (0.6, 1.5)	1.3 (0.8, 2.2)	1.3 (0.7, 2.3)
**Urbanicity (*n* = 656)**								
Urban	68.7	37.6	25.4	20.4	Referent	Referent	Referent	Referent
Rural	67.0	36.6	20.3	23.6	0.9 (0.5, 1.6)	0.8 (0.4, 1.3)	0.7 (0.3, 1.3)	1.0 (0.5, 2.0)
Suburban	73.3	41.7	23.6	24.7	1.3 (0.8, 2.0)	1.1 (0.7, 1.7)	0.9 (0.5, 1.4)	1.4 (0.8, 2.3)

* *p* < 0.05 across categories based on chi-squares tests. ^a^ Weight status was based on calculated body mass index (BMI) (kg/m^2^): underweight/healthy weight, BMI < 25; overweight, BMI 25–<30; obesity, BMI ≥ 30.

**Table 4:** Unadjusted (bivariate analysis) and adjusted (multivariate analysis) relationship between top four reasons why sugar-sweetened beverages (SSBs) are consumed according to sociodemographic variables among U.S. adult SSB consumers—Omnibus Survey, 2021.

Characteristics	Reasons Why SSB Are Consumed							
	Bivariate Analysis Prevalence (%)				Multivariate Logistic Regression Analysis Adjusted Odds Ratios (95% CI)			
	Taste	Craving	Fizz/bubbles	Thirst	Taste	Craving	Fizz/bubble	Thirst
**Age**								
18–34 years old	57.2	34.7 *	28.2 *	22.0	1.3 (0.8, 2.1)	2.8 (1.7, 4.8)	2.4 (1.4, 4.1)	1.3 (0.7, 2.3)
35–49 years old	63.4	35.0*	22.4 *	20.9	1.6 (1.0, 2.6)	2.8 (1.7, 4.9)	2.1 (1.2, 3.7)	1.2 (0.7, 2.1)
≥50 years old	51.8	20.4 *	14.8 *	18.7	Referent	Referent	Referent	Referent
**Gender**								
Men	56.1	27.3	20.0	21.6	Referent	Referent	Referent	Referent
Women	56.7	29.4	21.6	19.0	1.1 (0.8, 1.6)	1.1 (0.7, 1.7)	1.1 (0.7, 1.7)	1.0 (0.6, 1.6)
**Race/Ethnicity**								
Black, non-Hispanic	51.0	29.1	13.4	23.5	0.8 (0.5, 1.5)	0.8 (0.4, 1.6)	0.5 (0.2, 1.3)	1.2 (0.6, 2.5)
Hispanic	55.3	25.9	17.5	22.9	0.9 (0.5, 1.6)	0.7 (0.4, 1.3)	0.8 (0.4, 1.5)	1.1 (0.6, 2.0)
Other/multiracial, non-Hispanic	55.3	18.8	24.0	14.9	0.7 (0.4, 1.4)	0.4 (0.2, 1.0)	0.7 (0.3, 1.7)	0.7 (0.3, 1.8)
White, non-Hispanic	58.0	30.6	22.9	19.6	Referent	Referent	Referent	Referent
**Education**								
≤High school	52.7	28.0	19.3	22.1	0.7 (0.4, 1.1)	0.8 (0.5, 1.4)	0.5 (0.3, 1.0)	1.2 (0.7, 2.2)
Some college	54.8	28.9	21.4	22.1	0.6 (0.4, 1.0)	0.8 (0.5, 1.4)	0.7 (0.4, 1.2)	1.4 (0.8, 2.4)
College graduate	63.4	28.4	22.3	15.6	Referent	Referent	Referent	Referent
**Annual household income**								
<USD 25,000	38.7 *	26.9	27.1	21.4	0.6 (0.3, 1.1)	1.2 (0.5, 2.6)	2.7 (1.2, 6.1)	1.3 (0.6, 2.9)
USD 25,000–<USD 50,000	52.8 *	32.4	21.6	24.2	0.8 (0.5, 1.4)	1.9 (1.0, 3.6)	1.6 (0.8, 3.3)	1.5 (0.7, 2.8)
USD 50,000–<USD 100,000	61.1 *	30.0	21.5	20.5	1.1 (0.7, 1.7)	1.4 (0.8, 2.3)	1.7 (1.0, 3.0)	1.3 (0.8, 2.3)
≥USD 100,000	60.5 *	25.2	17.2	17.5	Referent	Referent	Referent	Referent
**Marital status**								
Not married	56.3	32.3	24.0	22.1	1.2 (0.8, 1.9)	1.1 (0.7, 1.8)	1.1 (0.7, 1.7)	1.1 (0.7, 1.8)
Married	56.5	25.5	18.4	18.9	Referent	Referent	Referent	Referent
**Weight status ^a^ (*n* = 602)**								
Underweight/healthy weight	58.8	24.9	18.6	16.4	Referent	Referent	Referent	Referent
Overweight	56.8	32.4	20.5	25.2	1.0 (0.7, 1.6)	1.8 (1.1, 3.0)	1.4 (0.8, 2.4)	1.7 (1.0, 3.1)
Obesity	55.0	29.3	20.7	22.2	1.0 (0.7, 1.6)	1.6 (1.0, 2.7)	1.4 (0.8, 2.5)	1.4 (0.8, 2.5)
**Census region**								
Northeast	60.3	34.7	18.4	16.2	1.3 (0.8, 2.2)	1.6 (0.9, 2.9)	1.2 (0.6, 2.3)	0.7 (0.4, 1.3)
Midwest	53.7	24.7	22.4	15.5	10 (0.6, 1.6)	0.7 (0.4, 1.3)	1.3 (0.7, 2.5)	0.6 (0.4, 1.2)
South	55.1	27.4	17.1	25.4	Referent	Referent	Referent	Referent
West	57.5	28.1	27.1	19	1.1 (0.7, 1.8)	1.2 (0.7, 2.1)	1.9 (1.1, 3.4)	0.7 (0.4, 1.3)
**Having children (<18 years old) in household**								
No	56.2	28.4	20.2	21.2	Referent	Referent	Referent	Referent
Yes	56.7	28.4	22.0	18.7	0.9 (0.6, 1.4)	0.8 (0.5, 1.4)	0.8 (0.5, 1.3)	0.8 (0.5, 1.4)
**Urbanicity (*n* = 656)**								
Urban	54.1	27.0	15.6	19.0	Referent	Referent	Referent	Referent
Rural	52.6	21.8	23.5	16.2	1.0 (0.6, 1.7)	0.7 (0.4, 1.4)	1.6 (0.8, 3.2)	0.7 (0.4, 1.5)
Suburban	59.3	31.9	22.7	22.7	1.4 (0.9, 2.1)	1.2 (0.8, 1.9)	1.6 (0.9, 2.8)	1.3 (0.8, 2.0)

* *p* < 0.05 across categories based on chi-squares tests. ^a^ Weight status was based on calculated body mass index (BMI) (kg/m^2^): underweight/healthy weight, BMI < 25; overweight, BMI 25–<30; obesity, BMI ≥ 30.

## Data Availability

The datasets generated and/or analyzed during the current study are not publicly available due licensing agreements but are available directly from the corresponding author on reasonable request.
